# Halogenated and metallic scaffolds as novel efflux pump inhibitors: mechanistic insight in overcoming antimicrobial and cross-domain resistance

**DOI:** 10.3389/fmicb.2026.1923157

**Published:** 2026-10-12

**Authors:** Manaf AlMatar, Raja Lakhal, Kamaleldin H. Kamaleldin, Arig Ibrahim-Hashim

**Affiliations:** 1 Department of Biology, Faculty of Education and Arts, Sohar University, Sohar, Oman; 2 Department of Engineering, University of South Florida, Tampa, FL, United States; 3 Massachusetts General Hospital Cancer Center, Harvard Medical School, Boston, MA, United States

**Keywords:** antimicrobial resistance (AMR), drug repurposing, efflux pump inhibitors (EPIs), halogen bonding, metal-based antimicrobial compounds, multidrug resistance (MDR)

## Abstract

Antimicrobial resistance (AMR) is increasing rapidly, necessitating the development of non-traditional therapeutic approaches. Overexpression of bacterial efflux pumps mediates active extrusion of drugs and is a major mechanism underlying multidrug resistance (MDR) in both Gram-negative and Gram-positive pathogens. Inhibition of efflux pumps (EPIs) can restore the activity of conventional antibiotics that have lost efficacy due to resistance. Here, we examine two classes of non-antibiotic adjuvants: halogenated small molecules and metal-based coordination complexes or nanoparticles. Halogen bonding and related chemical modifications can modulate membrane permeability and influence binding kinetics within bacterial transporters. Metal-based antimicrobial compounds utilize specific three-dimensional geometries and iron-mimicry to disrupt microbial transport systems. Notably, although microbial efflux pumps and mammalian multidrug transporters are structurally and mechanistically distinct, they share a functional role in reducing intracellular drug accumulation. This functional convergence provides a rationale for investigating whether selected EPI scaffolds developed in antimicrobial systems may also modulate resistance-associated transport processes in cancer. However, evidence supporting such cross-domain activity remains predominantly preclinical, and functional activity in one biological system should not be interpreted as evidence of therapeutic equivalence or clinical transferability to the other. By integrating structure–activity relationships, transporter mechanisms, microenvironmental pH effects, and translational limitations, this review highlights the potential of halogenated and metal-based scaffolds as experimental EPI platforms while emphasizing the substantial pharmacological, safety, and validation challenges that remain before clinical application.

## 1 Introduction

One of the most efficient and rapid ways bacteria develop resistance is through efflux pumps, which actively expel antibiotics and other toxic compounds (Li et al., 2015a; Du et al., 2018). Efflux pumps lower intracellular levels of drugs, making bacterial survival possible in otherwise fatal circumstances. These efflux transporters are divided into six major superfamilies: the ATP-binding cassette (ABC) family, the major facilitator superfamily (MFS), the multidrug and toxin extrusion (MATE) family, the small multidrug resistance (SMR) family, the resistance–nodulation–cell division (RND) superfamily, and the proteobacterial antimicrobial compound efflux (PACE) family (Du et al., 2018; Hassan et al., 2018). Overexpression of efflux pumps is an established cause of multidrug resistance (MDR) and hence inhibiting them is a highly promising therapeutic intervention (Blair et al., 2015).

Two novel classes of efflux pump inhibitors (EPIs) are halogenated compounds and metal-based antimicrobial compounds. Halogens are of critical significance in drug design, as approximately 25% of drugs on the market and 40% of research leads contain halogen atoms, with an extremely high proportion of blockbuster drugs being halogenated (Hernandes et al., 2010; Wilcken et al., 2013). Halogen incorporation can substantially alter the physicochemical and pharmacological properties of a molecular scaffold, but its effects are highly context dependent. Depending on the identity and position of the halogen and the characteristics of the parent scaffold, substitution may alter lipophilicity, membrane permeability, metabolic stability, electronic distribution, target affinity, and molecular conformation. These changes can improve, reduce, or have little effect on biological activity; therefore, the pharmacological consequences of halogenation should be evaluated on a scaffoldby-scaffold basis rather than assumed to be uniformly beneficial (Wilcken et al., 2013; Lu et al., 2009). These features have been utilized in the synthesis of halogenated antibiotics and antimicrobial scaffolds. Metal-based antimicrobial compounds, in contrast, leverage new three-dimensional geometries, redox character, and pleiotropic mechanisms of action (Frei, 2020; Lemire et al., 2013). Traditional antibiotics, whose mode of action is usually via one biochemical target, interfere with multiple cellular processes at the same time, and hence, it is more difficult for bacteria to become resistant. This pleiotropy, combined with potential synergy as adjuvants with other antibiotics, has revived interest in their therapeutic potential (Frei et al., 2020).

Crucially, overexpressed efflux pumps do not merely lower intracellular drug concentrations; they actively grant the bacterium a vital survival window needed to activate stress pathways, such as, the LexA-mediated SOS response, and acquire permanent genetic mutations (El Meouche and Dunlop, 2018; Kumawat et al., 2023; Baharoglu and Mazel, 2014; Cirz et al., 2005). Therefore, halogenated and metallic coordination complexes may function as promising next-generation inhibitors by suppressing this adaptive window.This bacterial transport machinery shares notable functional overlap with systems driving chemotherapy failure in cancer. Mammalian ABC transporters, such as P-glycoprotein (ABCB1), MRP1, and BCRP, actively efflux cytotoxic agents, thereby inducing tumor MDR (Robey et al., 2018; Gottesman et al., 2002). Interestingly, certain halogenated and metal-based EPIs developed initially against bacterial resistance, such as selenium-substituted flavonoids, gallium complexes, and halogenated alkaloids, inhibiting malignant cellular transporters as well (Kostova, 2024; Szakács et al., 2006). This functional overlap suggests that antibacterial EPI libraries may represent a valuable source of candidates for oncology-focused drug discovery.

However, the translational potential of these dual-acting EPIs must be carefully balanced against toxicity, bioaccumulation, and long-term safety issues. Future efforts must focus on robust structure–activity relationship (SAR) studies to optimize specificity, the engineering of biocompatible delivery systems such as nanoparticles, and the implementation of integrated screening platforms profiling efflux kinetics in both bacteria and cancer cells. Such multi-disciplinary strategies are poised to discover broad-spectrum EPIs with the definitive promise to enhance drug efficacy across both infectious and oncologic diseases (Figure 1).

**Figure 1:**
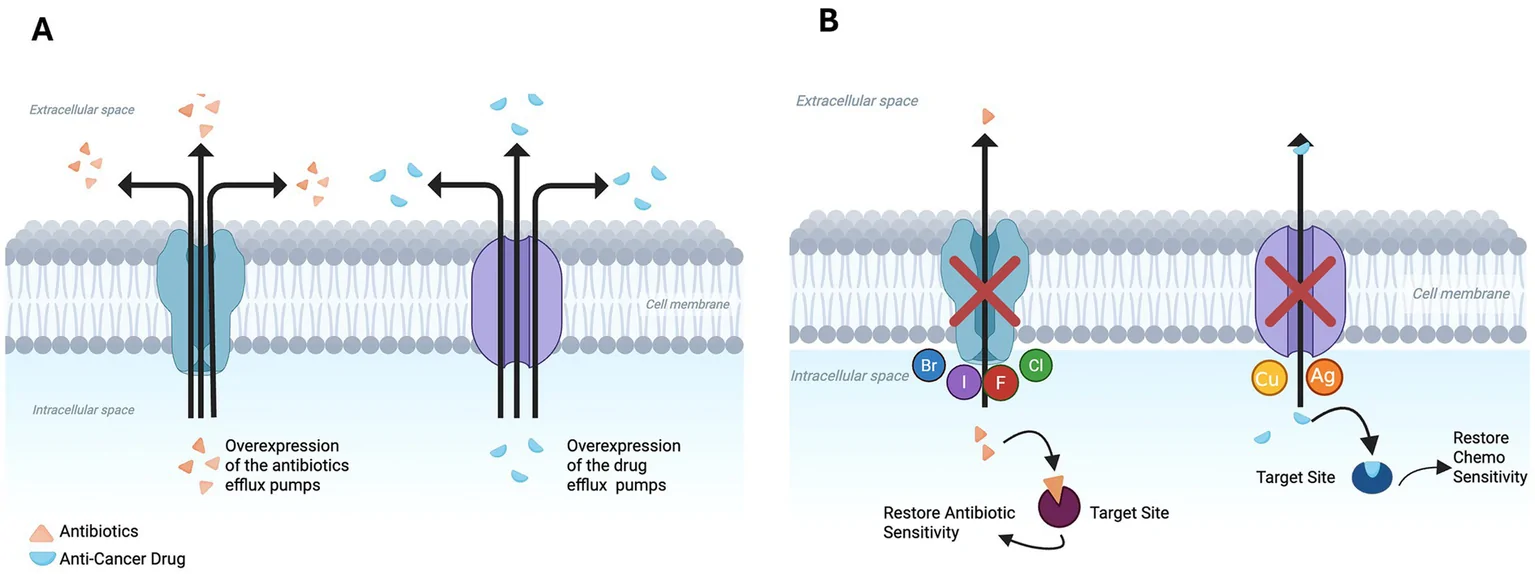
Schematic illustration of functional convergence and proposed therapeutic mitigation of efflux-mediated drug resistance across microbial and mammalian systems. **(A)** Overexpression of microbial efflux pumps can reduce intracellular antibiotic concentrations, while increased activity of mammalian drug transporters can similarly reduce intracellular retention of anticancer drug. **(B)** Halogenated compounds and metal-based systems have been investigated as potential modulators of these resistance-associated processes. The effects shown represent experimentally observed antimicrobial or transporter-associated activities in selected preclinical studies and should not be interpreted as evidence of structural or therapeutic equivalence between bacterial and mammalian transport systems. The cross-domain relationship is therefore presented as a functional analogy and hypothesis-generating framework, requiring direct experimental validation for therapeutic translation. Created with https://www.biorender.com/.

Accordingly, this review provides an integrated analysis of three interconnected but distinct areas: (i) halogenated natural and synthetic scaffolds as modulators of antimicrobial resistance and efflux-associated phenotypes, (ii) metal-based coordination compounds, nanoparticles, and siderophore-mediated systems as emerging antimicrobial and transporter-modulating strategies, and (iii) the extent to which these chemical approaches may inform resistance modulation in mammalian cancer systems. Rather than assuming mechanistic equivalence between bacterial and mammalian transporters, we critically evaluate the evidence for functional convergence, identify differences in transporter biology and pharmacology, and highlight the experimental and translational barriers that must be addressed before cross-domain EPI development can be considered clinically relevant.

## 2 Halogenated compounds

### 2.1 Halogenated natural and synthetic scaffolds with antimicrobial properties

Natural antimicrobials, derived from a wide range of sources including plants, animals, and both prokaryotic and eukaryotic organisms, have contributed substantially to the development of approved therapeutics (Quave, 2016). However, their effectiveness is often limited by narrow spectra of activity, low yields, enzymatic degradation, low toxicity, and the emergence of microbial resistance. A promising strategy to enhance or restore the pharmacological efficacy of both natural and synthetic compounds is the incorporation of halogens (Molchanova et al., 2020; Hurtová et al., 2020). As a result, recent research has increasingly focused on the use of halogenated compounds to combat emerging and re-emerging drug-resistant bacteria. This review explores the functional roles of these derivatives with respect to their chemical groups and structural stability. The compounds discussed include halogenated polyphenols, essential oils, alkaloids, benzopyrones, phenazines, azoles, repurposed drugs, and other bioactive molecules. Each section evaluates their efficacy against resistant infections, the role of halogenation, associated cytotoxicity, and underlying mechanisms of action (Figure 2).

**Figure 2:**
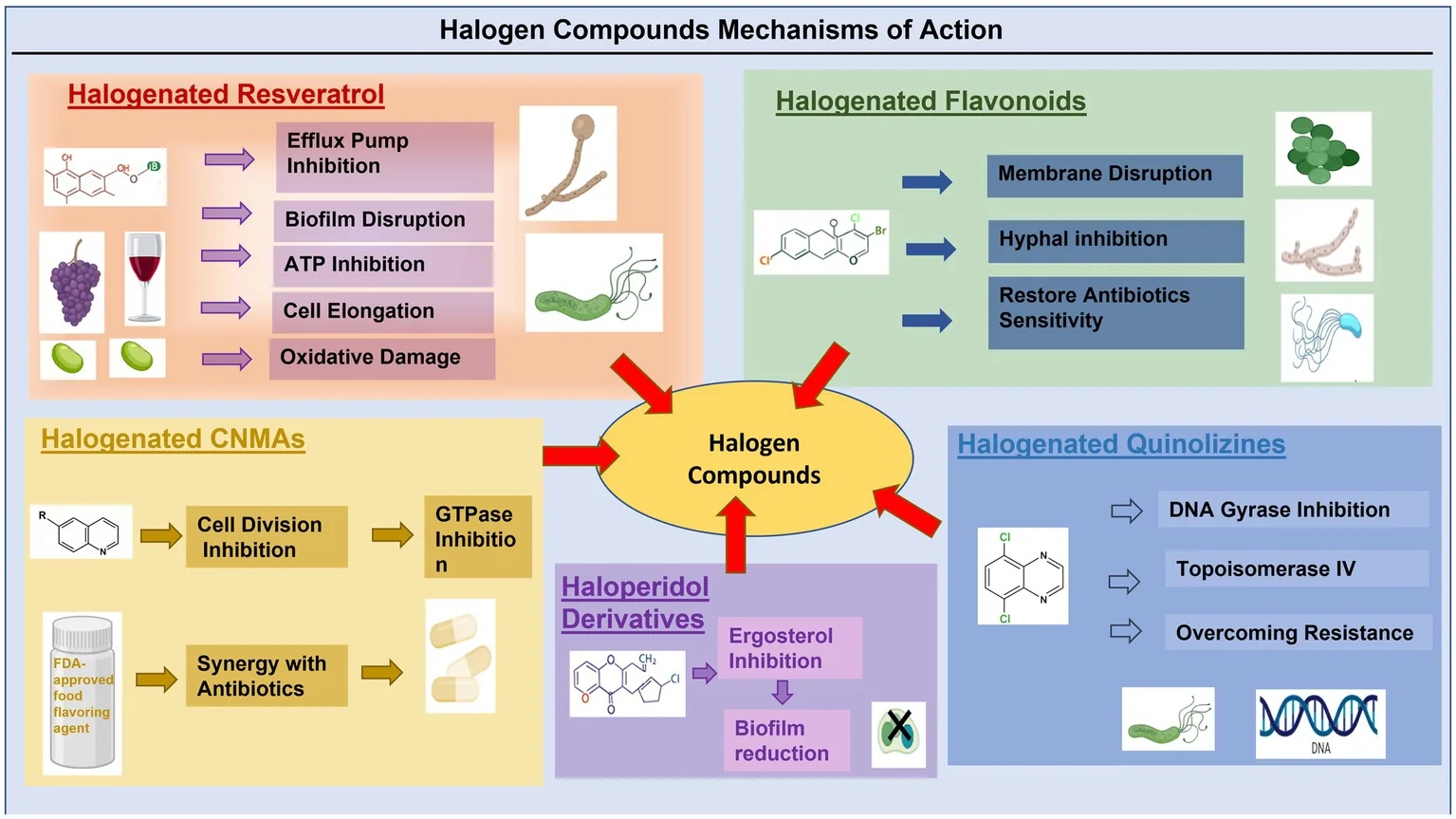
Proposed and experimentally supported antibacterial mechanisms of representative halogenated compounds. Halogenated resveratrol derivatives have demonstrated antimicrobial activity in preclinical studies through multiple reported effects, including efflux-associated modulation, biofilm disruption, ATP depletion, oxidative stress, and interference with bacterial cell division. Halogenated flavonoids have been reported to affect membrane integrity, hyphal formation, and antibiotic susceptibility. Halogenated cinnamaldehyde derivatives have been associated with inhibition of bacterial cell division and GTPase activity, whereas halogenated quinolizines primarily target essential bacterial enzymes, including DNA gyrase and topoisomerase IV. Haloperidol derivatives have demonstrated antifungal and efflux-associated effects in experimental models. Where direct transporter assays are unavailable, the mechanisms shown represent inferred or proposed explanations based on phenotypic, accumulation, gene-expression, or sensitization studies rather than definitive evidence of direct transporter inhibition. Most evidence summarized in this figure is derived from *in vitro* and other preclinical studies, and the mechanisms should not be interpreted as clinically validated. ATP, adenosine triphosphate; CNMAs, cinnamaldehyde derivatives.

#### 2.1.1 Scaffold dependence

The effects of halogenation on antimicrobial and efflux-associated phenotypes are strongly scaffold dependent. The biological consequences of introducing a halogen cannot therefore be generalized across chemically unrelated natural products, synthetic scaffolds, and repurposed drugs. Differences in molecular architecture, intrinsic target affinity, membrane permeability, metabolic stability, and susceptibility to bacterial efflux can determine whether halogenation improves antimicrobial activity, produces little change, or introduces new pharmacological liabilities. The following examples illustrate this scaffold dependence across polyphenols, flavonoids, cinnamaldehyde derivatives, quinolizine-based compounds, and repurposed pharmaceuticals.

Resveratrol, a phytoalexin with diverse biological and antibacterial properties, is naturally found in red wine, grapes, soybeans, mulberries, cranberries, and Japanese knotweed (Zhang et al., 2021; Vikal et al., 2024). Halogenated derivatives of resveratrol have demonstrated significant potential in overcoming antibiotic resistance (Di Fermo et al., 2020). Li et al. (2012) synthesized halogenated derivatives of resveratrol, of which 2-chloro-resveratrol and 2-bromo-resveratrol exhibited greater antifungal activity against *Candida albicans* than both the parent compound and fluconazole. In addition, 4-[E)-2-(4-chlorophenyl) ethenyl]phenol demonstrated excellent antibacterial activity against resistant *Helicobacter pylori (H. pylori)*, with MIC values of 3.1 μg/mL and 12.5 μg/mL, representing 64-fold and 16-fold improvements, respectively, over the parent compound (200 μg/mL) (Di Fermo et al., 2020). According to Vestergaard and Ingmer (2019) and Mattio et al. (2020), parent resveratrol exhibits limited efficacy against Gram-negative bacteria, primarily due to efflux pump mechanisms that hinder its accumulation at cytoplasmic or periplasmic targets (Vestergaard and Ingmer, 2019). However, a halogenated derivative of resveratrol has demonstrated the ability to inhibit these efflux systems and restore antibacterial activity. This effect was particularly evident in its synergistic interaction with levofloxacin, effectively suppressing two drug-resistant *H. pylori* strains at sub-MIC concentrations (Di Fermo et al., 2020). Targeting virulence factors presents a promising strategy for combating resistant infections. Sub- MIC of halogenated resveratrols have been demonstrated to disrupt *H. pylori* biofilm formation and motility key processes in gastric mucosa colonization (Di Fermo et al., 2020). These compounds display antibacterial activity across several experimental systems, with chlorine or bromine substitution associated with altered activity profiles relative to the parent scaffold. However, the contribution of the halogen itself cannot be separated from accompanying changes in molecular polarity, lipophilicity, conformation, and other structural features without systematic structure–activity and mechanistic studies. Halogenation boosts molecular lipophilicity, improving penetration into bacterial membranes and facilitating lipid peroxyl radical formation. Furthermore, halogenated resveratrols interfere with ATP synthesis and reduce bacterial adhesion, enhancing their antivirulence potential (Di Fermo et al., 2020). For instance, chlorinated resveratrol triggered cell elongation, indicating interference with cell division, and provided protective benefits in *Galleria mellonella* infected with *H. pylori* (Di Fermo et al., 2020). Moreover, this derivative showed no toxicity to *G. mellonella* larvae and lacked hemolytic activity against human red blood cells a property that could be influenced by the halogen atoms’ size or quantity. Previous structure activity studies of resveratrol analogues have highlighted the importance of hydroxyl substitution and molecular configuration for biological activity (Savio et al., 2009).

However, the pharmacological advantages of halogenation should be interpreted cautiously, as these effects are highly dependent on the chemical scaffold, halogen identity, and substitution position. Although halogen incorporation can improve metabolic stability and membrane permeability in selected compounds, increased lipophilicity may also adversely affect aqueous solubility, plasma protein binding, tissue distribution, and systemic exposure. In some cases, increased hydrophobicity may promote nonspecific interactions or prolonged tissue retention, potentially complicating clearance and safety assessment. Therefore, improved *in vitro* potency or metabolic stability of halogenated EPIs does not necessarily translate into improved *in vivo* efficacy or therapeutic index. Comprehensive evaluation of absorption, distribution, metabolism, excretion, protein binding, tissue distribution, and toxicity will be required before these compounds can be considered viable clinical adjuvants. These considerations are particularly important for halogenated EPIs because their therapeutic activity depends on achieving sufficient concentrations at bacterial or tumor sites while minimizing systemic and off-target exposure (Hernandes et al., 2010; Walle, 2011; Bourgault et al., 2011; Summerfield and Pattison, 2026).

Halogenated flavonoids, including BrCl-flav and ClCl-flav, demonstrate antibacterial effects by inducing intracellular content leakage, elevating membrane permeability to biocides, and distorting cellular structure (Babii et al., 2021; Babii et al., 2018). Compound 8 J also exhibits antifungal activity against *Candida* spp. By causing irreversible morphological changes and blocking hyphal formation (Babii et al., 2021). The bactericidal potency of compound 8 K is further amplified by halogen substitution at the C4 position and the presence of a 1,3-dithiolium ring (Babii et al., 2018). Additionally, flavonolignan 8-bromo-2,3-dehydrosilybin AB, when combined with antibiotics at breakpoint concentrations, was found to restore colistin sensitivity in resistant *P. aeruginosa* strains. Similarly, 6,8,21-tribromosilybin A and 6,8,21tribromosilybin B showed potent activity against gentamicin-resistant *Staphylococcus aureus* (*S. aureus*) at 40 μM (Hurtová et al., 2022). Bromination of flavonolignans substantially boosts antibacterial efficacy and counteracts resistance mechanisms, likely through efflux pump inhibition, disruption of bacterial signaling, and improved selectivity (Hurtová et al., 2022).

The antimicrobial activity of Cinnamomum species is largely due to trans-cinnamaldehyde (CNMA), an FDA-approved food flavoring agent (Shreaz et al., 2016; Friedman, 2017). Halogenating CNMA has emerged as a viable approach to enhance its effectiveness against antibiotic-resistant bacteria. Studies show that 2,4-dichloro- and fluorinated CNMA derivatives exhibit superior activity against penicillin-resistant *Streptococcus pyogenes* and *S. aureus*, with MIC values of 16–64 μg/mL—significantly lower than those of cinnamic acid and ciprofloxacin (MIC >128 μg/mL) against resistant *S. pyogenes* (Li et al., 2015b). These halogenated derivatives (R and S) also inhibited drug-sensitive *S. aureus* at much lower concentrations (4–16 μg/mL). Surprisingly, drug-susceptible *S. pyogenes* showed higher tolerance (MIC ≥128 μg/mL), requiring roughly double the dose needed for resistant strains, suggesting the presence of a resistance mechanism targeted by these compounds. Further modifications have yielded even more potent derivatives. For instance, p-brominated CNMA displayed an MIC of 0.51 mM against *S. aureus* and *Escherichia coli* (*coli. E. coli*), making it 4–5 times more effective than unmodified CNMA (1.93–2.32 mM) (Doyle et al., 2019). Additionally, 4-bromophenyl-substituted CNMA and 2,4-dichlorinated CNMA (were active against MRSA and *Acinetobacter. Baumannii* (*A. baumannii*) (64 μg/mL) but ineffective against extensively drug-resistant *A. baumannii* unless combined with an EPI, implying efflux-mediated resistance (Chai et al., 2022). The intrinsic resistance of *A. baumannii* is partly due to its highly impermeable outer membrane, which lacks nonspecific porins, reducing permeability ~100-fold compared to *E. coli* (Chai et al., 2022; Zgurskaya et al., 2015). To improve activity against multidrug-resistant *A. baumannii*, further halogen modifications to the CNMA scaffold may be necessary. Mechanistically, halogenated CNMAs disrupt bacterial growth by interfering with cell division. They induce cell elongation, inhibit FtsZ polymerization, and impair GTPase activity, ultimately leading to defective cytokinesis and cell death (Li et al., 2015b; Chai et al., 2022).

The 4H-4-oxoquinolizine scaffold, a quinolone bioisostere, was designed to overcome the drawbacks of conventional fluoroquinolones, including limited activity against Gram-positive bacteria and increasing resistance (Ma et al., 1999). Halogenated 4-oxoquinolizine derivatives exhibit stronger antibacterial effects than fluoroquinolones. Notably, compounds CC195776, CC-195767, and CC-195820 displayed exceptional potency against drug-resistant *A. baumannii*, with MICs as low as 0.09–0.19 μg/mL, far surpassing ciprofloxacin, ofloxacin, and levofloxacin (MICs: 8–64 μg/mL) (Na et al., 2017). Another promising candidate, GC072, currently in preclinical phase, demonstrated superior efficacy against drug-resistant *Burkholderia pseudomallei*, with MICs ranging from ≤0.004 to 0.25 μg/mL, outperforming ciprofloxacin (0.25–16 μg/mL). Additionally, TNP-2092, a prodrug combining a 4H-4quinolizine pharmacophore with a rifamycin moiety, showed strong activity against resistant *H. pylori* (MICs: 0.008–16 μg/mL), exceeding the performance of clarithromycin and levofloxacin (0.016–128 μg/mL) (Wang et al., 2018). Fluoroquinolone resistance typically arises from mutations in the quinolone resistance-determining regions (QRDRs) of *GyrA and ParC* (Madurga et al., 2008). However, halogenated 4-oxoquinolizines retain binding affinity for these mutated enzymes, maintaining activity against resistant strains. For example, CC-195776, CC195767, and CC-195820 remained effective against *A. baumannii* with QRDR mutations (Na et al., 2017). Similarly, GC-072 outperformed ciprofloxacin against trimethoprim- and ceftazidime-resistant strains, likely due to mutations in *folA* and *PenA β-lactamase* (Shearer et al., 2019). Resistance was further mitigated by inhibiting efflux pumps. While GC-072 resisted AmrAB-OprA and BpeAB-OprB-mediated efflux in *B. pseudomallei*, it remained partially susceptible to BpEF-OprC, though far less than ciprofloxacin and doxycycline. Like fluoroquinolones, halogenated 4-oxoquinolizines target DNA gyrase and topoisomerase IV, but with enhanced selectivity and a unique binding mode (Shearer et al., 2019). Toxicity studies revealed that CC195776, CC-195767, and CC-195820 were non-toxic to human HeLa and U937 cells at 25 μg/mL, though cytotoxicity emerged at 50 μg/mL— ~ 100-fold higher than their MICs (0.09–0.19 μg/mL) (Na et al., 2017). Overall, halogenated 4H-4-oxoquinolizines demonstrate greater efficacy than fluoroquinolones in combating resistant bacteria, making them a promising alternative.

Haloperidol, a butylbenzoic antipsychotic drug, undergoes primary hepatic elimination via glucuronidation and CYP3A4-mediated oxidation, with high plasma protein binding and extensive liver metabolism (Kudo and Ishizaki, 1999). Beyond its neurological applications, haloperidol has demonstrated antifungal properties, particularly against fluconazole-resistant *C. albicans*. A fluorinated derivative (compound 11D) exhibited enhanced antifungal activity, inhibiting fungal growth at 32 μg/mL, significantly lower than fluconazole’s MIC of >64 μg/mL. C4position fluorine substitution on the terminal benzene ring was associated with enhanced antifungal activity and may influence the metabolic and pharmacological properties of the derivative compared with haloperidol (Ji et al., 2019). The haloperidol derivative 11D (4-fluorophenyl haloperidol) disrupts fungal pathogenesis by suppressing key virulence factors. It significantly diminishes biofilm formation, melanin production, urease activity, and capsular polysaccharide synthesis, critical components for cell wall integrity, immune evasion, and pathogenicity in *Cryptococcus neoformans*, thereby influencing host survival and disease progression (Ji et al., 2019; Revankar and Sutton, 2010; O’Meara and Alspaugh, 2012). When combined with fluconazole, compound 11D reduced the MIC to 4 μg/mL and prevented resistance development in fluconazole-resistant *C. albicans* by downregulating the efflux pump gene and inhibiting ergosterol biosynthesis (Ji et al., 2019). Similarly, bromoperidol derivatives synergized with posaconazole or voriconazole, overcoming resistance in azole-resistant *C. albicans* strains (Matos et al., 2018; Holbrook et al., 2017). These findings highlight the potential of haloperidol-derived compounds as antifungal adjuvants, particularly by targeting drug transporters like MDR, aligning with previous research (Iwaki et al., 2006). Importantly, the antifungal concentrations reported for haloperidol-derived compounds should not be equated with clinically achievable haloperidol exposure. Compound 11D demonstrated antifungal activity at 32 μg/mL *in vitro*, whereas therapeutic haloperidol concentrations in clinical use are typically measured in the ng/mL range. Pharmacokinetic studies indicate substantial interindividual variability in haloperidol exposure, with clinical effects occurring at plasma concentrations far below the micromolar-to-tens-of micrograms-per-milliliter concentrations used in antimicrobial experiments. Therefore, the observed antifungal and efflux-modulating activities of haloperidol-derived compounds should be regarded as preclinical findings rather than evidence that conventional neurological dosing of haloperidol would provide clinically relevant antifungal or efflux inhibitory exposure. Translation of these findings will require dedicated dose–response studies, pharmacokinetic characterization, safety evaluation, and determination of whether therapeutically effective antimicrobial concentrations can be achieved without unacceptable neurological or systemic toxicity (Kudo and Ishizaki, 1999; Ji et al., 2019; Osacka et al., 2022; Coryell et al., 1998).

#### 2.1.2 Structure–activity relationships and mechanistic considerations

Across these chemically diverse scaffolds, the antimicrobial consequences of halogenation appear to depend on both the parent structure and the identity and position of the introduced halogen. Fluorine, chlorine, bromine, and iodine differ in electronegativity, polarizability, steric demand, and their effects on lipophilicity and electronic distribution. Consequently, halogen substitution may alter membrane partitioning, target interactions, molecular conformation, metabolic stability, and transporter recognition in a scaffold-dependent manner. The cinnamaldehyde and flavonoid studies illustrate this principle, as different halogen substitutions and substitution positions produce distinct antimicrobial activity profiles rather than a uniform increase in potency. Thus, the presence of a halogen should not be considered independently from the underlying scaffold or substitution pattern when interpreting structure–activity relationships (Hernandes et al., 2010; Hurtová et al., 2022; Doyle et al., 2019).

From a medicinal-chemistry perspective, fluorine generally introduces relatively little steric bulk while exerting substantial electronic effects, whereas chlorine and bromine provide progressively greater steric volume and polarizability and can produce larger changes in lipophilicity. Iodine has the greatest polarizability and steric contribution among these substituents and may therefore introduce additional hydrophobicity and formulation or metabolic liabilities. These differences can influence membrane partitioning, target binding, transporter interactions, antimicrobial potency, and toxicity. However, the available evidence does not support a universal potency ranking of F, Cl, Br, and I because the effect of each substituent depends strongly on the molecular scaffold and substitution position. Comparative studies within the same chemical series are therefore more informative than comparisons across unrelated scaffolds.

Importantly, increased antimicrobial potency does not necessarily correspond to improved therapeutic potential. The 4H-4-oxoquinolizine series provides an illustrative example: selected compounds showed MIC values of 0.09–0.19 μg/mL against resistant *A. baumannii*, while cytotoxicity toward HeLa and U937 cells emerged at approximately 50 μg/mL, indicating a substantial experimental separation between antibacterial and cytotoxic concentrations (Na et al., 2017). However, such selectivity margins should be interpreted cautiously because cytotoxicity assays, exposure times, cell types, and antimicrobial endpoints differ among studies. A more meaningful assessment of therapeutic potential therefore requires standardized concentration–response analyses, mammalian-cell cytotoxicity, and, where relevant, hemolytic and organ-specific toxicity assessments.

#### 2.1.3 Pharmacological and translational limitations

Despite promising *in vitro* activity, the translational potential of halogenated scaffolds remains constrained by pharmacokinetic, formulation, and safety considerations. Halogenation can improve metabolic stability in selected compounds, but these effects are highly scaffold and substitution dependent. Increased lipophilicity may, in some cases, increase plasma protein binding, alter tissue distribution, reduce aqueous solubility, or contribute to prolonged tissue retention and accumulation. Conversely, other halogenated compounds may undergo rapid metabolism or fail to achieve adequate systemic exposure despite favorable antimicrobial potency *in vitro*.

Resveratrol illustrates these challenges. Although resveratrol has demonstrated broad biological activity, its clinical development is limited by poor bioavailability, rapid metabolism, instability, and the formation of inactive metabolites (Vikal et al., 2024; Walle, 2011; Francioso et al., 2014). Selected halogenated resveratrol analogs have demonstrated improved stability or pharmacological activity in experimental models, suggesting that halogen substitution may partially address some limitations of the parent scaffold. However, the effects of halogenation on absorption, distribution, metabolism, and clearance remain compound dependent, and improved experimental stability or biological activity should not be interpreted as evidence of superior clinical pharmacokinetics without direct comparative exposure data.

These considerations are particularly important for efflux-pump inhibitors because therapeutic activity depends on achieving sufficient concentrations at the relevant bacterial or tumor site while minimizing systemic and off-target exposure. Formulation and delivery strategies may also be critical for poorly soluble or highly lipophilic candidates. Therefore, improvements in MIC, intracellular accumulation, or transporter-associated activity should not be interpreted as evidence of therapeutic superiority unless supported by quantitative pharmacokinetic, biodistribution, safety, and *in vivo* efficacy data.

### 2.2 Classes of halogenated antibiotics

Antibiotics play a vital role in chemotherapy by targeting bacterial and fungal infections. The twentieth-century discovery of penicillin by Alexander Fleming marked a turning point in modern medicine, leading to the development of numerous other antibiotics (Fleming, 2001). However, their excessive and often improper use has contributed to the rise of drug-resistant pathogens, underscoring the pressing need for novel antimicrobial agents (Pulingam et al., 2022). Halogens have been integral to antibiotic development since the identification of chloramphenicol in 1947, appearing in various antibiotic classes and core scaffolds. Moreover, halogenated metabolites are found in diverse natural sources, such as microbes, algae, plants, and animals (Hutchings et al., 2019). Antibiotics are typically classified by chemical structure, mechanism of action, or spectrum of activity. In this analysis, we focus on halogenated antibiotics grouped by their mechanisms of action, particularly where the halogen directly contributes to antimicrobial efficacy or helps combat drug-resistant strains. Despite the high potency of fluoroquinolones, bacteria have evolved resistance mechanisms, including reduced membrane permeability, increased efflux, and target-site mutations. Strategies such as efflux pump inhibition or enhancing membrane permeability can counteract this resistance. For instance, halogenated EPIs have improved the efficacy of halogenated fluoroquinolone adjuvants against *S. aureus* NorA and MepA efflux pumps (Freitas et al., 2021), as well as halogenated pyridylpiperazine-based inhibitors targeting *E. coli* RND-type efflux pumps (Plé et al., 2022). Among halogenated lincosamides, clindamycin and pirlimycin stand out (Spížek and Řezanka, 2017), with chlorine substitution at the 7-carbon position significantly boosting clindamycin’s activity. Notably, *E. coli* is 20 times more sensitive to clindamycin than to lincomycin, highlighting the impact of halogenation (Douthwaite, 1992). Lincosamides and macrolides share resistance mechanisms, such as efflux pumps and ribosomal modifications. To address these challenges, researchers have developed macrolones, a hybrid class combining macrolide and quinolone structures. Halogenated macrolone derivatives exhibit strong activity against multidrug-resistant *E. coli* and *S. pneumoniae* (Fajdetić et al., 2010; Aleksandrova et al., 2024; Čipčić Paljetak et al., 2016). Specifically, halogenated analogs of 4”-O-(ω-quinolylamino-alkylamino) propionyl macrolides effectively inhibit resistant strains of Streptococcus spp., Staphylococcus spp., and *Haemophilus. influenzae* (Fajdetić et al., 2010). These findings suggest that halogenated compounds may overcome common resistance mechanisms, including efflux pumps and ribosomal alterations, offering a promising avenue for combating resistant bacteria.

Taken together, these observations indicate that the contribution of halogens to antibiotic performance extends beyond simple chemical substitutions. Halogen incorporation repeatedly improves antimicrobial performance through multiple overlapping mechanisms including improved membrane permeability, enhanced target affinity, reduced susceptibility to transporter-mediated extrusion, and restoration of activity against resistant strains. Importantly, these effects appear across multiple antibiotic classes, suggesting that halogenation may serve as a transferable design principle capable of extending the lifespan of existing antimicrobial scaffolds.

### 2.3 How halogenation affects antibiotic resistance mechanisms

The strength of evidence supporting efflux inhibition varies substantially among the compounds discussed. Direct evidence includes biochemical transporter assays, binding studies, structural characterization, or experiments demonstrating inhibition of a defined efflux pump independently of changes in general membrane permeability. Intermediate evidence may include increased intracellular antibiotic accumulation accompanied by genetic or pharmacological evidence implicating a specific transporter. In contrast, restoration of antibiotic susceptibility, increased inhibition-zone diameter, or suppression of resistance-associated gene expression alone does not establish direct transporter inhibition because these phenotypes may also result from membrane disruption, altered metabolism, stress responses, or other off-target mechanisms. Accordingly, the compounds reviewed here are distinguished as having direct transporter evidence, indirect efflux-associated evidence, or primarily phenotypic evidence.

Halogenation improves the penetration of antimicrobial agents through cell membranes and optimizes their ADME (absorption, distribution, metabolism, and excretion) properties. This chemical modification can also revitalize antimicrobials that have lost effectiveness against drug-resistant strains, reestablishing their antibiotic susceptibility.

Halogenated compounds counteract multiple resistance mechanisms, including efflux inhibition, membrane disruption, ATP depletion, oxidative stress induction, biofilm inhibition, and interference with bacterial cell division (Figure 2). Among these mechanisms, efflux pumps which expel antibiotics before they reach their intracellular targets are a major contributor to resistance and a key therapeutic focus (Nishino et al., 2021). Halogenated compounds effectively combat resistance by blocking efflux pumps. For instance, 3,4-dibromopyrrole-2,5-dione inhibits overexpressed RND pumps (AcrAB-TolC, MexAB-OprM, and MexXY-OprM), reversing multidrug resistance in *E. coli* to antibiotics like ciprofloxacin, levofloxacin, and tetracycline (Mahey et al., 2024; Whalen et al., 2015). Similarly, ethyl 4-bromopyrrole2-carboxylate restores antibiotic susceptibility in multidrug-resistant *E. coli* and *P. aeruginosa* by suppressing RND transporters when combined with antibiotics (Tambat et al., 2022). Proton-dependent efflux pumps are another target of halogenated compounds. The association between trifluoromethyl substitution and proton-pump inhibition remains a structure activity hypothesis rather than an established causal mechanism, because direct biochemical evidence linking the CF₃ group itself to transporter inhibition is currently limited. Additionally, halogenated 1,3,5-triphenyl-2-pyrazolines inhibit bacterial efflux pumps in *M. tuberculosis* and other pathogens, reducing antimicrobial resistance (Balganesh et al., 2012). Halogenated compounds can also downregulate efflux pump genes, thereby diminishing drug expulsion. Trifluoromethyl ketones, for example, inhibit proton pumps, enhancing the effectiveness of ampicillin, tetracycline, and erythromycin against resistant *E. coli* strains. Likewise, 3-(2-benzoxazolyl)-1,1,1-trifluoro-2-propanone inhibits *E. coli* proton pumps, enhancing the activity of promethazine (Wolfart et al., 2006; Kawase et al., 2001). Chlorpromazine, for example, improves norfloxacin’s efficacy against *Salmonella enterica* by suppressing the *acrB* gene, reducing drug export and increasing intracellular accumulation. Similarly, a haloperidol derivative reverses fluconazole resistance in *C. albicans* by inhibiting the MDR efflux pump gene (Ji et al., 2019). These findings highlight the synergistic potential of halogenated compounds and antibiotics in combating efflux-mediated resistance. Using halogenated adjuvants alongside antibiotics could thus be a viable strategy to restore drug efficacy against multidrug-resistant (MDR) strains. By reducing drug expulsion, increasing intracellular antibiotic concentrations, and directly inhibiting pathogens, halogenated compounds offer a promising approach to overcoming antimicrobial resistance.

Overall, evidence from diverse halogenated compounds supports a multi-mechanistic model through which resistance can be overcome. Rather than targeting a single pathway, these molecules frequently combine direct transporter inhibition, modulation of membrane permeability, suppression of efflux-associated gene expression, and restoration of intracellular antibiotic accumulation. This mechanistic redundancy may represent a significant advantage, potentially reducing the likelihood that pathogens can rapidly evolve resistance through single adaptive pathways. However, mechanistic redundancy may also complicate therapeutic development. Compounds that simultaneously perturb efflux, membrane integrity, metabolism, oxidative balance, and stress-response pathways may produce off-target toxicity and make it difficult to attribute efficacy to a specific molecular target. Multi-target activity can also complicate structure– activity optimization because improving one pharmacological property may adversely affect another. Therefore, mechanistic redundancy should be considered a potential advantage for overcoming resistance but also a development liability requiring target deconvolution, selectivity profiling, and systematic toxicity assessment. The principal mechanisms and levels of supporting evidence across these compounds are summarized comparatively in Table 1.

**Table 1:** Comparative mechanisms and evidence supporting efflux-associated and antimicrobial activity of representative example.

Mechanism	Representative examples	Main evidence (level of evidence)	Functional outcome	Interpretation
Direct efflux inhibition	CoHex	Direct transporter assay (direct/high)	TolC inhibition	Stronger mechanistic evidence than phenotypic sensitization alone
Efflux-associated modulation	ZnO-NPs, AgNPs, CuNPs	Gene-expression/phenotypic (indirect/moderate)	Resistance modulation	Supports efflux involvement but does not necessarily establish direct transporter binding
Membrane disruption	Halogenated flavonoids, metal NPs	Cellular assays (phenotypic/indirect)	Increased permeability	May increase drug accumulation independently of efflux
ATP/energy disruption	Halogenated resveratrol	Metabolic/phenotypic (indirect/moderate)	Reduced energy-dependent resistance	Indirect evidence of efflux involvement
Increased intracellular accumulation	PAA-magnetite NPs	Accumulation assay (indirect/moderate)	Increased antibiotic exposure	Consistent with impaired efflux but not definitive
Biofilm/virulence suppression	Resveratrol derivatives, 11D	Phenotypic (phenotypic/low for EPI)	Reduced biofilm/virulence	Distinct from direct EPI activity
Target-specific antibacterial activity	4-oxoquinolizines, CNMA derivatives	Target/mechanistic studies (mechanistic; not EPI evidence)	Gyrase/FtsZ inhibition	Not necessarily efflux-mediated

## 3 Metal-based antimicrobial agents and uptake-associated resistance mechanisms

Hexaamminecobalt (III) (CoHex) acts as an EPI by binding tightly to the TolC protein in *E. coli* at 0.74 μM (Gilardi et al., 2017) (Figure 3A). However, despite blocking TolC function, CoHex lacks antibacterial properties and does not enhance antibiotic effectiveness. In comparison, certain metal-based nanoparticles (metallo-NPs) can suppress efflux pump gene expression. For instance, thiosalicylic acid-functionalized ZnO@Glu-TSC nanoparticles inhibit efflux pumps and exhibit antibacterial effects against ciprofloxacin-resistant *S. aureus*, with MICs between 8 and 512 μg/mL. When paired with ciprofloxacin, ZnO@GluTSC boosts antibacterial activity 32-fold by downregulating key resistance genes (*norA, norB, norC, tet38*, Wang et al., 2025). Similarly, ZnO nanoparticles (ZnO-NPs) hinder the NorA efflux pump in *S. aureus*, increasing ciprofloxacin’s inhibition zone by 27%, and also combat *A. baumannii* (MIC = 31 μg/mL) by altering *AdeB* and *AdeRS* gene expression. Thiolated chitosan-coated cobalt-doped ZnO-NPs further enhance antibacterial action (MIC = 10 μg/mL) by disrupting *S. aureus* efflux mechanisms (Iqbal et al., 2019). Silver nanoparticles (AgNPs) suppress multiple efflux genes (*AdeA, AdeC, AdeS, AdeR, AdeI, AdeJ, AdeK*) in drug-resistant *A. baumannii* (MIC = 25–200 μg/mL) and inhibit efflux in *B. pseudomallei* (MIC = 32 μg/mL) (Behdad et al., 2020). Copper nanoparticles (CuNPs) target the NorA pump in *S. aureus*, and when combined with ciprofloxacin, reduce its MIC 4-fold (from 20 μM to 5 μM) (Christena et al., 2015). Additionally, poly (acrylic acid)-coated magnetite nanoparticles (PAA-MNPs) inhibit efflux in *Mycobacterium smegmatis*, cutting rifampicin’s MIC 4-fold (40 μM to 4 μM) and tripling intracellular drug accumulation (Padwal et al., 2014). These findings highlight metallo-NPs as promising EPIs that can rejuvenate antibiotic efficacy against resistant bacteria. Further research is needed to refine their design and evaluate clinical potential in overcoming AMR. Despite their promising antimicrobial and efflux-modulating activities, the translational application of metal-based nanoparticles requires careful consideration of their safety and long-term biological behavior. Metal nanoparticles can interact with cellular membranes, proteins, mitochondria, and nucleic acids and may induce reactive oxygen species (ROS) generation, oxidative stress, inflammation, mitochondrial dysfunction, apoptosis, and other off-target effects. Importantly, nanoparticle toxicity is highly dependent on physicochemical characteristics, including particle size, morphology, surface charge and functionalization, composition, aggregation state, dissolution behavior, and dose, as well as the route and duration of exposure. For copper-based nanoparticles, dissolution and release of Cu ions may contribute substantially to oxidative stress, cytotoxicity, genotoxicity, and inflammatory responses. *In vivo* studies have also demonstrated that copper oxide nanoparticles can undergo tissue distribution and accumulation, particularly within organs involved in the mononuclear phagocyte system, although biodistribution varies according to nanoparticle composition and surface properties. Silver nanoparticles likewise require careful evaluation because their biological activity and toxicity may be influenced by particle size, surface characteristics, dose, and the release of silver ions. Therefore, the therapeutic development of silver-, copper-, and other metal-based EPIs should incorporate systematic assessment of pharmacokinetics, biodistribution, cellular and organ toxicity, clearance, and potential accumulation following repeated or systemic exposure. Such studies are essential to determine whether the antimicrobial benefits of these materials can be achieved within a clinically acceptable therapeutic window (Wang et al., 2024; Sajjad et al., 2023; Thomas et al., 2024; Dey et al., 2019).

**Figure 3:**
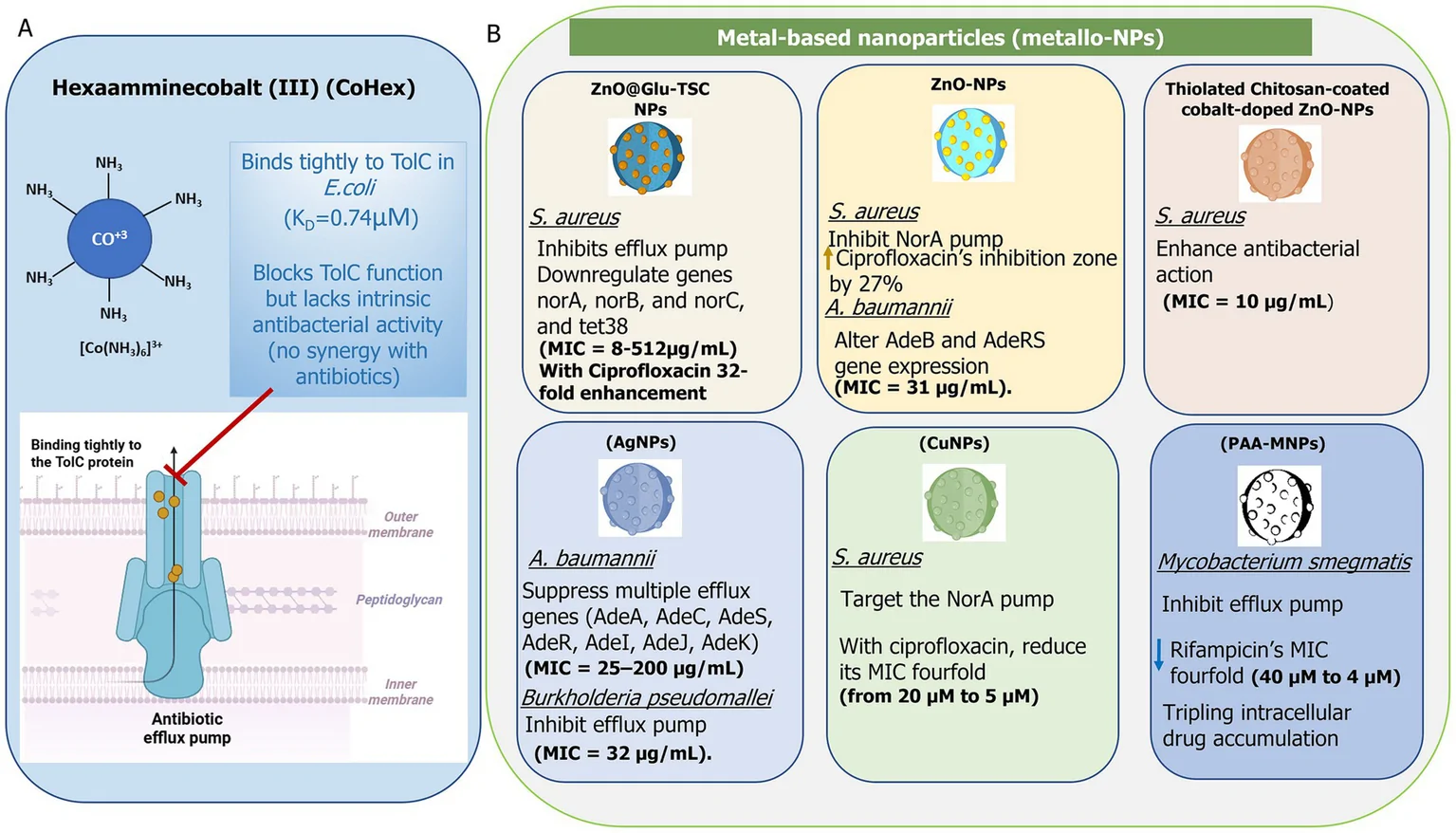
Representative metal-based antimicrobial compounds and nanoparticles investigated for efflux-associated and antimicrobial activity. **(A)** Hexaamminecobalt(III) (CoHex) has been shown experimentally to bind the TolC outer membrane channel of *Escherichia coli* and inhibit efflux pump function. **(B)** Metal-based nanoparticles, including ZnO@Glu-TSC nanoparticles, ZnO nanoparticles, cobalt-doped ZnO nanoparticles, silver nanoparticles (AgNPs), copper nanoparticles (CuNPs), and polyacrylic acid-coated magnetic nanoparticles (PAA-MNPs), have demonstrated antimicrobial, efflux-associated, or resistance-gene-modulating effects in experimental bacterial models. Reported effects include modulation of NorA and AdeABC-associated systems, changes in resistance-associated gene expression, and increased intracellular antibiotic accumulation. The mechanisms and therapeutic effects shown are predominantly supported by *in vitro* or other preclinical studies; where direct transporter inhibition has not been demonstrated, the mechanisms are presented as proposed or inferred rather than established. The figure does not imply clinical validation of these metal-based systems. MIC, minimum inhibitory concentration; TolC, outer membrane efflux channel protein.

From a translational perspective, metal-based systems differ substantially in their development challenges. Soluble metal complexes and siderophore-conjugated agents may offer more predictable composition and pharmacokinetic behavior than particulate systems, although their development remains dependent on metal stability, ligand exchange, systemic exposure, and metal-associated toxicity. In contrast, metal-based nanoparticles offer opportunities for controlled delivery and surface functionalization but present additional challenges because particle size, morphology, surface chemistry, aggregation, dissolution, and protein corona formation can influence their stability, biodistribution, clearance, and biological activity. Silver- and copper-based nanoparticles may release biologically active metal ions, potentially increasing oxidative and inflammatory toxicity, whereas iron- and zinc-based systems may exhibit different degradation or dissolution profiles but should not be presumed to be inherently safer. Importantly, biodegradability, dissolution, and clearance must be established experimentally for each formulation rather than inferred from elemental composition alone. Regulatory translation may also be more challenging for nanoparticle products because consistent control of particle size, composition, surface characteristics, aggregation state, impurities, and manufacturing processes are required. Thus, antimicrobial potency alone is insufficient to establish translational readiness; comparative assessment of stability, biodistribution, biodegradation or dissolution, clearance, toxicity, manufacturing reproducibility, and regulatory feasibility is necessary before metal-based EPIs can be considered clinically viable (Bobo et al., 2016; Blanco et al., 2015).

Representative metal-based EPIs and nanoparticles are summarized in Figure 3B. Iron is an essential micronutrient for microbes, acting as a cofactor in many enzymes that facilitate crucial biological functions. However, its low solubility and fierce competition in host environments make iron acquisition especially in the ferric (Fe^3+^) state a key factor for bacterial survival (Kramer et al., 2020). To address this, bacteria produce siderophores: small molecules (400–2,000 Da) that tightly bind Fe^3+^, allowing them to scavenge iron from their surroundings and sustain metabolic processes (Lin et al., 2025). These siderophores form highly stable Fe^3+^ complexes using functional groups like catecholates, hydroxamates, or carboxylates. Siderophore drug hybrids, or sideromycins, can significantly boost antibiotic potency sometimes by hundreds of times (Schalk, 2018). Researchers are particularly interested in metal-based sideromycins because bacteria preferentially absorb siderophores, reducing drug toxicity in human cells. One innovative approach involves using siderophores to deliver metal-containing antibiotics, improving effectiveness especially against Gramnegative bacteria while sparing human cells. For instance, a ruthenium (III)-siderophore complex has shown antibacterial effects against pathogens like *E. coli* and *P. aeruginosa* with minimal toxicity to human cells (IC₅₀ > 200 μM). In contrast, cisplatin, a potent anticancer drug, is too toxic for broad antibacterial use. To circumvent this, a “Trojan horse” strategy was developed, using enterobactin to deliver Pt (IV) drugs specifically into bacterial cells, reducing human cell uptake (~0.01%) (Daghistani et al., 2025; Szymańska et al., 2025). This approach could repurpose metal-based drugs by leveraging siderophores for targeted delivery. Since enterobactin binds Fe (III) tightly, researchers have explored other metals with similar properties to create synthetic sideromycin complexes. For example, Gabofloxacin a Ga (III)-ciprofloxacin–desferrichrome conjugate—shows diagnostic and antibacterial activity against *P. aeruginosa* and *S. aureus*, with MIC as low as 0.093μMv (Pandey et al., 2021; Pandey et al., 2019). Similarly, a salmochelin–ciprofloxacin hybrid demonstrated strong antibacterial effects, especially when radiolabeled with Ga (III) (Pandey et al., 2019). Additionally, combining Bi(III) with cefiderocol enhanced its potency against resistant *P. aeruginosa*, reducing the MIC from 16 μM to 1 μM (Wang et al., 2023). This synergy was also effective in mouse models, suggesting clinical potential. In conclusion, metal–siderophore conjugates improve antibacterial performance against resistant strains while reducing toxicity. These metallo-sideromycins could prolong the effectiveness of existing antibiotics, warranting further exploration of different siderophores and metals to combat AMR. Despite their attractive uptake mechanism, siderophore-mediated delivery is subject to several biological limitations (Negash et al., 2019). Siderophore receptor expression varies substantially among bacterial species and strains and may change during infection or under different nutrient conditions (Diarra et al., 1996). Genetic alteration, downregulation, or loss of the cognate receptor can therefore reduce uptake and generate resistance to siderophore–drug conjugates (Galdino et al., 2024). In addition, siderophore pathways are embedded within complex host–pathogen iron competition, and host iron-binding proteins may influence the availability, trafficking, and uptake of siderophore complexes (Wilson et al., 2016; Steigedal et al., 2014). These considerations indicate that the effectiveness of siderophore-mediated delivery depends not only on metal affinity but also on receptor abundance, substrate specificity, bacterial physiology, and the capacity of pathogens to alter iron-acquisition pathways.

Collectively, metal-based antimicrobial systems provide mechanistic advantages that extend beyond conventional efflux pump inhibition. Coordination complexes, functionalized nanoparticles, and siderophore-mediated delivery systems introduce structural diversity, redox activity, and transporter-targeting capabilities that may circumvent traditional resistance mechanisms. Importantly, many of these systems operate through multiple simultaneous mechanisms including transporter disruption, altered gene expression, membrane perturbation, and targeted uptake pathways supporting their development as multifunctional antimicrobial adjuvants. A comparative summary of representative halogenated and metal-based EPI candidates, including their targets, supporting evidence, quantitative outcomes, principal advantages, and major translational limitations, is provided in Table 2.

**Table 2:** Comparative summary of representative halogenated and metal-based antimicrobial resistance-modulating candidates, including efflux pump inhibitors and related resistance-modulating systems.

Scaffold/compounds	Targets	Mechanism & evidence	Quantitative outcome	Advantage	Limitation/stage
Resveratrol derivatives2-Cl-resveratrol; 2-Br-resveratrol	*H. pylori*	Efflux-associated; phenotypic	MICs 3.1–12.5μg/mL	Synergy; antivirulence	Poor PK; *in vitro*/limited *in vivo* (Di Fermo et al., 2020; Li et al., 2012; Vestergaard and Ingmer, 2019; Savio et al., 2009; Walle, 2011)
Halogenated flavonoids: BrCl-flav; tribromosilybins	*P. aeruginosa*, *S. aureus*	Membrane/efflux-associated; phenotypic	40μM	Antibiotic resensitiza-tion	Direct EPI evidence limited; *in vitro* (Babii et al., 2018; Hurtová et al., 2022)
Cinnamaldehyde derivatives	*S. aureus*, *A. baumannii*	FtsZ/cell division; mechanistic	MICs 16–64μg/mL	Improved potency	Scaffold-dependent; *in vitro* (Shreaz et al., 2016; Friedman, 2017; Li et al., 2015a; Doyle et al., 2019; Chai et al., 2022; Zgurskaya et al., 2015)
4-Oxoquinolizines: CC-195776; GC-072	*A. baumannii*, *B. pseudomallei*	Gyrase/topoisomerase; mechanistic	MICs 0.09–0.19μg/mL	High potency	Limited clinical validation; preclinical (Ma et al., 1999; Na et al., 2017; Wang et al., 2018; Madurga et al., 2008; Shearer et al., 2019)
Haloperidol derivatives: 11D	*C. albicans*	Efflux/virulence modulation; phenotypic	MIC 32μg/mL; 4μg/mL + fluconazole	Azole synergy	Clinical exposure concern; *in vitro* (Ji et al., 2019; Revankar and Sutton, 2010; O’Meara and Alspaugh, 2012; Matos et al., 2018; Holbrook et al., 2017)
CoHex	*E. coli* TolC	Direct efflux-channel binding	Binding 0.74μM	Direct target evidence	Direct target evidence (Gilardi et al., 2017)
ZnO nanoparticles	*S. aureus*, *A. baumannii*	Efflux/membrane effects; phenotypic	32-fold CIP enhancement	Resistance reversal	Toxicity/PK/regulatory issues; *in vitro* (Section 3 references)
Ag nanoparticles	*A. baumannii*	*A. baumannii*	MICs 25–200μg/mL	Broad activity	Ion release/toxicity; *in vitro* (Section 3 references)
Cu nanoparticles	*S. aureus*, NorA	Efflux modulation; phenotypic	CIP MICs 20→5μM	4-fold sensitization	Cu toxicity/accumulation; *in vitro* (Section 3 references)
Magnetite NPs	*M. smegmatis*	Efflux/accumulation; phenotypic	RIF MICs 40→4μM	Increased accumulation	Clearance/formulation; *in vitro* (Section 3 references)
Siderophore systems	*E. coli*, *P. aeruginosa*	Iron-uptake exploitation; functional	IC50 >200μM	Selective uptake strategy	Receptor-dependent; *in vitro*

## 4 Therapeutic convergence: dual-utility EPIs in antimicrobial resistance and cancer multidrug resistance

Efflux-mediated resistance represents a major obstacle in both antimicrobial therapy and cancer chemotherapy. In both settings, transporter proteins reduce intracellular drug concentrations and thereby limit therapeutic efficacy. Although bacterial multidrug transporters and mammalian ATP-binding cassette (ABC) transporters differ substantially in structure and energy utilization, they share functional similarities as multidrug extrusion systems. These functional parallels have stimulated interest in compounds capable of modulating resistance mechanisms across biological systems. Importantly, these similarities should be interpreted as functional convergence rather than structural equivalence. Bacterial multidrug efflux systems comprise several transporter families with distinct architectures and mechanisms, including the major facilitator superfamily (MFS), resistance–nodulation–cell division (RND), multidrug and toxic compound extrusion (MATE), small multidrug resistance (SMR), and bacterial ATP-binding cassette (ABC) transporters. Many bacterial MFS, RND, and MATE systems use electrochemical ion gradients, predominantly the proton motive force, to drive substrate export, whereas ABC transporters couple drug translocation to ATP binding and hydrolysis. In contrast, mammalian multidrug resistance is prominently mediated by ABC transporters such as P-glycoprotein (ABCB1), MRP1 (ABCC1), and BCRP (ABCG2), which use ATP hydrolysis to power conformational changes and active substrate extrusion. Bacterial RND systems may additionally form large tripartite assemblies spanning the inner membrane, periplasm, and outer membrane, a structural organization that has no direct equivalent in mammalian ABC drug transporters. Consequently, although both bacterial and mammalian systems can decrease intracellular drug concentrations and promote therapeutic resistance, their differences in molecular architecture, substrate-recognition mechanisms, membrane organization, and energy coupling represent important barriers to direct cross-domain translation. The identification of compounds with activity in both systems should therefore be regarded as a hypothesis-generating strategy requiring direct biochemical, structural, cellular, pharmacokinetic, and toxicity validation rather than evidence of transporter equivalence (Alcalde-Rico et al., 2016; Siasat and Blair, 2023; Murakami, 2008; Thomas and Tampé, 2020; Chen et al., 2016). In particular, halogenated small molecules and metal-based antimicrobial compounds have emerged as promising candidates because of their ability to alter membrane permeability, interfere with transporter activity, and enhance intracellular drug accumulation (Li et al., 2015a; Robey et al., 2018).

### 4.1 Halogenated flavonoids and alkaloids

Halogen substitution is widely employed in medicinal chemistry because it can increase lipophilicity, membrane permeability, metabolic stability, and target-binding affinity. Flavonoids have attracted considerable attention as EPIs due to their ability to modulate both bacterial transporters and mammalian ABC proteins. Several halogenated flavonoid derivatives have demonstrated antibacterial or efflux-associated activity, while separate studies of structurally related flavonoids have reported modulation of mammalian ABC transporters. These observations provide a hypothesis-generating rationale for investigating cross-domain activity, but do not establish that the same halogenated compounds inhibit both transporter systems (Gwenaelle et al., 1998).

### 4.2 Metal-based antimicrobial compounds

Metal-containing compounds represent another structurally diverse class of potential dual-purpose EPIs. Selenium-containing molecules, including selenium-substituted flavonoids, have demonstrated the ability to inhibit mammalian ABC transporters while simultaneously exhibiting antimicrobial activity, potentially owing to their redox properties and interactions with membrane-associated proteins (Spengler et al., 2019).

Gallium (III) complexes are metal-based drugs that disrupt microbial iron uptake by mimicking ferric iron, which impairs bacterial growth especially in drug-resistant strains. They also show anti-cancer activity and affect pathways linked to drug resistance. Gallium-based antimicrobials, including gallium nitrate and experimental gallium–ligand compounds, have been evaluated in preclinical models, while clinical experience with gallium-containing agents provides additional pharmacological and safety context (Guo et al., 2023; He et al., 2023; Scott et al., 2022).

Despite promising properties, several issues limit gallium’s clinical use. Large trials proving benefits in severe bacterial infections are lacking, and unformulated Ga^3+^ breaks down quickly in the body and cannot be given by mouth, requiring ongoing IV treatment. Data on new oral and inhaled gallium formulations in humans are limited. High iron levels or abnormal transferrin saturation in severe illness may reduce gallium’s effectiveness. The safe dose and risks in people with kidney disease are not fully understood (Chitambar, 2012; Al-Gizawiy et al., 2024; Truong et al., 2023).

Nanoparticle-based systems further broaden the potential applications of metal-containing EPIs. Silver nanoparticles and zinc oxide nanoparticles have demonstrated antibacterial activity partly through modulation of efflux systems and membrane disruption, Separately, nanotechnology-based systems have been investigated as approaches to improve intracellular drug delivery and retention in chemo-resistant tumor models (Zhang et al., 2022).

Collectively, these findings suggest that functional similarities between microbial and mammalian multidrug transport systems may provide opportunities for cross-domain therapeutic development. Although significant translational challenges remain, including toxicity, pharmacokinetics, and transporter selectivity, the ability of certain halogenated compounds and metal-based systems to influence resistance mechanisms in multiple biological contexts supports further exploration of dual-purpose EPIs.

### 4.3 Translational limitations and barriers

Despite the mechanistic rationale for dual-purpose EPIs, the current evidence remains predominantly preclinical and several barriers limit translation into clinical applications. First, pharmacokinetic behavior may differ substantially among chemical classes, with poor aqueous solubility, rapid metabolism, extensive plasma protein binding, limited tissue penetration, or inadequate exposure potentially preventing effective concentrations from being achieved at the intended site of action. Second, transporter selectivity remains a major challenge because inhibition of mammalian ABC transporters may alter the disposition of concurrently administered drugs and produce clinically relevant drug–drug interactions. Third, metal-based compounds and nanoparticles introduce additional considerations, including biodistribution, tissue accumulation, degradation or dissolution, clearance, immunological effects, and long-term toxicity. Formulation is also particularly important for poorly soluble halogenated compounds and nanoparticle systems because particle size, surface chemistry, aggregation, and manufacturing conditions can influence stability, biological distribution, and reproducibility. Finally, regulatory development requires reproducible manufacturing, well-defined composition and impurity profiles, validated pharmacokinetic and toxicological assays, and demonstration of a favorable therapeutic index. Thus, enhanced antimicrobial or efflux-inhibitory activity *in vitro* should be regarded as an early-stage indicator rather than evidence of clinical utility. Future development should prioritize compounds demonstrating a combination of direct transporter activity, selective pharmacology, favorable ADME properties, reproducible formulation, and validated *in vivo* efficacy and safety.

## 5 Microenvironmental pH dynamics: bridging bacterial infection sites and the tumor microenvironment

A critical, yet frequently underrecognized, determinant of EPI efficacy and pharmacokinetic stability is the localized physiological microenvironment, specifically the marked extracellular acidification characteristic of both bacterial infection niches and solid tumors. While extracellular acidosis in malignancy (pH_e 6.5–6.8) is primarily driven by accelerated glycolytic flux via the Warburg effect and compromised vascular perfusion (Gillies et al., 2012; Webb et al., 2011), localized bacterial infection sites, including chronic urinary tract infections (UTIs), deep-tissue abscesses, and dense bacterial biofilms, similarly exhibit heavily acidic microenvironments (Schaffer and Pearson, 2015; Stewart and Franklin, 2008). In these infectious niches, accumulated bacterial metabolic byproducts, hypoxia, and host immune cell infiltrates drive a localized drop in pH that profoundly impacts transporter kinetics and inhibitor bioactivity (Stewart and Franklin, 2008; Lardner, 2001).

Importantly, class modulator EPIs, such as phenylalanine-arginine\beta-naphthylamide (PA\betaN) or carbonyl cyanide m-chlorophenylhydrazone (CCCP), alongside various novel transporter modulators, demonstrate highly variable, context-specific behaviors that are strictly dependent on the localized pH of the target environment. Fluctuations in pH alter the protonation states of both the transport proteins and the inhibitors themselves, potentially modulating binding affinity, membrane permeability, and pump-binding kinetics (Li et al., 2015a; Lomovskaya et al., 2001).

This microenvironmental parallel provides a seamless mechanistic transition when evaluating dual-utility therapeutic platforms: the identical pH-responsive dynamics and proton-motive force variations that govern metal-based and halogenated EPI kinetics within acidic bacterial infection sites may provide a conceptual framework for investigating analogous pH-dependent behavior in the acidic tumor microenvironment. Consequently, characterizing how these novel halogenated structures and coordination complexes respond to altered proton concentrations not only refines their development as potent anti-infective adjuvants but also optimizes their targeted, context-selective activation within the acidic landscapes of malignant tissues.

Specific examples of pH-responsive metal coordination systems further illustrate how acidity can be exploited to modulate therapeutic activity. Metal–ligand coordination bonds can undergo protonation-dependent weakening or dissociation under acidic conditions, thereby altering complex stability, ligand exchange, molecular conformation, or drug release. For example, pH-responsive metal–supramolecular systems incorporating coordination interactions have been developed to remain relatively stable under physiological conditions while undergoing structural reorganization or enhanced cargo release under acidic conditions. Similar principles have been explored using metal– organic frameworks and other coordination-based platforms, in which changes in metal– ligand interactions under acidic conditions can regulate molecular release and biological activity. These systems provide a useful conceptual framework for EPIs because acidic bacterial infection niches and tumor microenvironments may provide localized conditions capable of modifying coordination equilibria. However, pH responsiveness alone does not establish selective therapeutic activity, and the stability, ligand-exchange kinetics, metal release, and biological behavior of each complex must be evaluated under physiologically relevant pH, ionic strength, protein, and serum conditions (Wang et al., 2026; Amarsy et al., 2022; Ruan et al., 2024).

## 6 Conclusion

Antimicrobial resistance (AMR) and oncological multidrug resistance (MDR) represent two of the most critical global health and economic hazards of the modern biomedical era. This review has comprehensively evaluated the profound potential of next-generation halogenated scaffolds and metal-based coordination complexes to disrupt efflux-mediated resistance a shared molecular “gatekeeper” mechanism driving therapeutic failure in both microbial pathogens and malignant cells.

Strategically integrating halogens into foundational antibacterial architecture leverages unique halogen bonding mechanics, ultimately optimizing molecular lipophilicity, modifying membrane permeability, and significantly boosting binding affinity within poly-specific efflux cavities. Concurrently, metal-based antimicrobial compounds, metal coordination complexes, and functionalized nanoparticles introduce distinct three-dimensional geometries, redox-active properties, and multi-target mechanisms of action—such as iron-mimicry pathways that make the evolutionary acquisition of microbial resistance exceptionally difficult.

Prokaryotic transport networks and mammalian ABC transporters exhibit a functional convergence in mediating drug efflux and promoting therapeutic resistance, yet are distinguished by substantial differences in protein architecture, substrate recognition, membrane orientation, and modes of energy transduction. These mechanistic disparities present significant translational challenges, underscoring the necessity for direct experimental interrogation before bacterial EPI libraries can be rationally repurposed for cancer therapy. The identification of chemical scaffolds capable of modulating resistance pathways in both bacterial and mammalian systems establish a mechanistic basis for hypothesis-driven drug development. Notably, the extracellular acidification observed in both bacterial biofilms and tumor microenvironments represent a shared physiological feature that may be leveraged for the design of pH-responsive therapeutic adjuvants. The clinical translation of such strategies will depend on a comprehensive evaluation of candidate compounds, including their coordination chemistry, pH-dependent stability, ligand-exchange kinetics, and release dynamics under physiologically relevant conditions. Although pre-clinical studies of halogenated and metal-based EPIs have demonstrated promising activity, these agents remain largely restricted to *in vitro* or early-stage *in vivo* models. Rigorous future investigations should prioritize standardized efficacy metrics, detailed toxicity assessment, pharmacokinetic profiling, and systematic translational validation.
